# Cosmopolitanism and foreign policy for health: ethics for and beyond the state

**DOI:** 10.1186/1472-698X-13-29

**Published:** 2013-07-08

**Authors:** Raphael Lencucha

**Affiliations:** 1 Faculty of Medicine, School of Physical and Occupational Therapy, McGill University, Hosmer House, 3630 Promenade Sir William Osler, Montréal, QC H3G 1Y5, Canada

## Abstract

**Background:**

Foreign policy holds great potential to improve the health of a global citizenship. Our contemporary political order is, in part, characterized by sovereign states acting either in opposition or cooperation with other sovereign states. This order is also characterized by transnational efforts to address transnational issues such as those featured so prominently in the area of global health, such as the spread of infectious disease, health worker migration and the movement of health-harming products. These two features of the current order understandably create tension for truly global initiatives.

**Discussion:**

National security has become the dominant ethical frame underlying the health-based foreign policy of many states, despite the transnational nature of many contemporary health challenges. This ethical approach engages global health as a *means* to achieving national security objectives. Implicit in this ethical frame is the version of humanity that dichotomizes between “us” and “them”. What has been left out of this discourse, for the most part, is the role that foreign policy can play in extending the responsibility of states to protect and promote health of the *other, for the sake of the other*.

**Summary:**

The principal purpose of this paper is to review arguments for a cosmopolitan ethics of health-based foreign policy. I will argue that health-based foreign policy that is motivated by security interests is lacking both morally and practically to further global health goals. In other words, a cosmopolitan ethic is not only intrinsically superior as a moral ideal, but also has potential to contribute to utilitarian ends. This paper draws on the cosmopolitanism literature to build robust support for foreign policies that contribute to sustainable systems of global health governance.

## Background

National health indicators present a troubling picture of health disparity between nations. The life expectancy in Canada is 32 years longer than that of Afghanistan [1]. The chances of a child dying before reaching the age of 5 years in 2009 was 161/1000 live births in Angola versus 5/1000 live births in Australia [1]. In Bangladesh only 18% of births are attended by a skilled health professional, whereas in Italy, 99% of births receive such care [1]. Inter-governmental initiatives such as the Millennium Development Goals have garnered attention to global health disparities and have mobilized efforts to narrow the health gap between nations [2]. However, mechanisms to implement initiatives that would then work towards achieving these goals are often funded unilaterally and are periodic and absent measures of multilateral accountability to how the funds are spent [3]. Foreign development assistance is often fragmented and vulnerable to the shifting economic conditions of the donor country making the actual monetary commitments unreliable at best and in extreme circumstances withheld entirely. Global health initiatives are challenging, in part, because there are limited mechanisms to hold independent states accountable, leaving some states vulnerable while others hold unequal power. Supranational mechanisms of coordination do not presently exist, creating a complex environment of voluntary bi- and multi-lateral engagement among nations. Although the World Health Organization has acquired a dominant normative role in global health governance, the intergovernmental nature of the organization makes it vulnerable to the individual interests of states. It is precisely this context of inter*national* engagement that will set the context for this discussion of the ethics of foreign policy for health.

Given the often transnational features of global health it is fair to ask whether state-based ethical perspectives are acceptable and sufficient to achieve global health goals? What remains unchallenged in the foreign policy for health discourse is the assumption that state citizenship *should* be the locus of moral concern. This approach assumes that national governments are ultimately responsible to their citizens before they are responsible to any other group of persons, a “morality of states” [4]. This emphasis on a responsibility to state citizenship is reflected in the security frame that has come to dominate health-based foreign policy. This frame fosters a view that sees global health as a *means* to achieving national security objectives. In other words, a security frame is motivated by the notion that “we will help you if it enhances our own interests at home”, interests that will protect and prefer the well being of a politically bounded group of persons over global persons. What has been left out of this same discourse, for the most part, is the role that foreign policy can play in extending the responsibility of states to protect and promote health of the *other, for the sake of the other*, a cosmopolitan perspective. This perspective is not new to the arena of international affairs and is reflected in the Universal Declaration of Human Rights and the International Covenant on Economic, Social and Cultural Rights. However the ideal, and indeed the potential to manifest this perspective has yet to gain traction as a dominant paradigm for a global order.

What separates health from many other state interests ^a^ is that 1) health pertains to individual well-being and is widely accepted as a basic human right, and 2) health is profoundly influenced by social, economic and political conditions but should not be conflated with these conditions. In other words health is an individual phenomenon and should be recognized as such.^b^ To protect and promote health is to protect and promote the right of the individual, a basis individual right. From this characterization of health, one can differentiate legitimate health security threats from those that are not security threats by asking the question, is this threat a threat to the individual or to the state? Now this criterion is not always clearly discernible given that threats to a state can also be threats to individual health and vice versa. For example, bioterrorism may be directed towards a political entity but utilizes threats to the health of individuals. However, it would take a creative imagination to characterize chronic disease as a threat to the state while at the same time recognizing that interstate arrangements contribute to the conditions whereby individuals acquire and treat chronic disease (e.g. in the promotion of unhealthy products, or the rules that impact medical resources to manage chronic conditions).^c^

The ethical challenge for those who develop foreign policy for health is compounded by the observation that “virtually no systematic efforts have emerged to deal with moral foundations of *global* (emphasis added) health” (p. 427) [5,6]. Despite the paucity of ethical reasoning about global health, human rights scholars and practitioners have done exceptional work to ensure that individual rights are protected within the intra- and inter-state apparatus. The United Nations International Covenant on Economic, Social and Cultural Rights marked a victory to further embed the right to health in international law. However, the lack of universal support for such agreements coupled with the ability of states to enforce the social and economic conditions that foster individual health across borders remains a predominant challenge.

The principal purpose of this paper is to present arguments for a cosmopolitan ethics of foreign policy for health. Cosmopolitanism places the individual (and subsequently persons) as the primary unit of moral concern. In other words, I will argue that foreign policy based on charity or security is lacking both morally and practically to further global health goals. The ethical motivations underlying foreign policy for health are numerous and at times conflicting [5]. This paper begins by describing four common ethical perspectives underlying health-based foreign policy. In describing the four perspectives I will argue that the third ethical perspective (security) has become entrenched in the global health discourse. In the remainder of the paper I will argue 1) that this perspective is morally lacking and 2) that the fourth perspective, one motivated by cosmopolitanism, is not only morally superior (it is right) but is better suited to achieve the goals of foreign policy for health (it is good). I conclude this paper by arguing that not only is a cosmopolitan ethical frame morally superior and better suited to the achievement of global health, but also it is possible within the contemporary global political, economic and social order. This project is dedicated to bolstering a dialogue that recognizes that “the power of ethical values and notions of human solidarity should not be underestimated” [7].

## Discussion

### A typology of ethical perspectives to foreign policy

Countries interact and engage with the world in alignment with their foreign policy, which is motivated by different intentions [8,9]. The following section identifies and describes a typology of ethical approaches to health-based foreign policy to better situate and understand the scope and site of a cosmopolitan ethics (See Table 1).

**Table 1:** A typology of ethical perspectives

**Typology**	**Characteristics**
**Isolationism**	Extreme self-interest
	Self-otherness (Very high)
	State-centered
**Charity**	Self-otherness (High)
	Reactive
	Aide-oriented support
	State-centered
**Security**	Self-otherness (High)
	Reactive
	Long-term support/development
	Aide-oriented support
	State-centered
**Cosmopolitanism**	Self-otherness (Low)
	Long-term support
	Systems development
	Person-centered

### Isolationism

The first ethical perspective is the simplest. This perspective is characterized by extreme self-interest. The state-based identity is dominant for this perspective, to the extent that interaction with the “other” is inconsequential, antagonistic or non-existent. The best example of this perspective is the foreign policy of North Korea. This type of foreign policy is in fact no or minimal foreign policy for health. Health goals are entirely inward and reflect an extreme form of self-interest.

### Charity

The second perspective is perhaps the most common. This perspective is motivated by ‘charity’. Charity engenders a foreign policy that motivates voluntary, periodic engagement with events where an imminent threat to the health of the “other” is present. This ethical perspective is easily observed in the foreign policy of states following natural disasters or catastrophic events. Recent examples include the devastating earthquakes in Haiti, Pakistan, Japan, and New Zealand.

The support that results from this type of foreign policy is often not coordinated with other states, is temporary and reactive. For example, Merchant and colleagues recently described the complex coordination, or rather primarily uncoordinated efforts, that were involved in the post-disaster response to the 2010 earthquake in Haiti [10]. The response to the earthquake in Haiti provides a pointed and common example of a charity-based response to a natural disaster. This response is characterized by hundreds of non-governmental organizations (NGOs) such as Doctors without Borders, Partners in Health and the Red Cross working alongside, not necessarily in collaboration or coordination with, governments and intergovernmental organizations. Michaelle Jean, the former Governor General of Canada and a native to Haiti commented that the much of the efforts in Haiti have not delivered sustainable programming and have been carried out independent of the wishes of the Haitian government. The following statement reaches to the heart of the challenge of a charity-based ethical frame:

“Right now, the government of Haiti is completely decapitalized. Even after the earthquake, of all the money and the financial commitments of the international community to support the reconstruction of Haiti, only one per cent went to the government of Haiti.

Now the government cannot compete with the NGOs on the ground, which can pay a lot more for very skilled workers. That creates a total contradiction. The donor countries have validated the Haitian strategic plan for reconstruction but are not supporting the government in its capacity to implement those policies. It doesn’t make sense [11]”.

Although many circumstances call for this type of response from governments and other actors, the above example demonstrates the limitations of this approach to provide sustainable measures.

Pressing global issues such as the rise in non-communicable diseases (NCDs) are often neglected by a charity perspective simply because of the orientation to short-term relief efforts that are often not coordinated with the host government. To address NCDs on a global scale, a commitment to the development of long-term governance mechanisms that address complex, systemic determinants such as the international trade of health-harming products is required [12]. A charity-based foreign policy can support preparation for disaster preparedness but it tends to create reactive forms of engagement rather than the development of ongoing system strengthening [13]. The premise of this ethical perspective, at least for governments, is that “we take care of the health of our nation on a sustained basis, but if others need help in the occurrence of an emergency then we will help until the emergency is resolved”. This type of engagement tends to be voluntary and rendered as much by NGOs as by states.

### Security

The third ethical perspective reflects a country’s decision to act for the protection and promotion of the health of “the other” for the sake of their own citizens. This type of engagement has taken over from the charity perspective as the dominant ethical frame in the foreign policy for health discourse [14]. National security has historically been at the top of the foreign policy hierarchy [15]. It is in light of this history that it is not surprising that legislators and health advocates have attempted to frame health issues in security terms. The security rhetoric is found in such high level programs as the US Global Health Initiative. At the launch of this initiative on May 5th, 2009, then Secretary of State Hillary Clinton noted that the initiative would be “a crucial component of American foreign policy and a signature element of smart power” [16]. Deputy Secretary of State Jack Lew further expounded on the security rhetoric a few days after the launch of the initiative by stating: “we have the opportunity to cost-effectively contribute to political stability in a way that enhances our national security, while advancing our core humanitarian values” [17]. More pointedly, national-interest finds itself embedded in two of three of the United States’ Department of Health and Human Services global health strategy goals related in late 2011 [18]. Goal 1 is to protect and promote the health and well being of Americans through global health action. Goal 3 is to advance the United States’ interests in international diplomacy, development and security through global health action. These examples reflect the dominant inward emphasis taken by decision-makers. This inward focus on “us” is reasonable given the order of sovereign states and the pressures imposed within states to rationalize “global” spending. It is true that states face numerous fiscal and political realities that draw them into the protection and promotion of domestic interests. However, this paper argues that the narrow emphasis on security (as well as charity and non-engagement) may be antithetical to global health objectives.

Despite the recent dominance of the security perspective scholars have begun to highlight the over-securitization of health issues in the development of foreign policy [19]. Katz and Singer note, “health issues that do not pose security threats should not be contextualized as such, since doing so may detract from overarching public health and foreign policy objectives” (p. 233) [20]. For example, the commitment made by the United States to the global HIV/AIDS pandemic through the President’s Emergency Plan for AIDS Relief (PEPFAR) was deeply rooted in national security interests [15]. For example, the National Intelligence Council (NIC) produced a report titled The Global Infectious Disease Threat and Its Implications for the United States wherein it is stated that “These diseases (including HIV/AIDS) will endanger US citizens at home and abroad, threaten US armed forces deployed overseas, and exacerbate social and political instability in key countries and regions in which the United States has significant interests” [21]. Although it is tempting to assert that the emergence of PEPFAR indicates that a security frame can indeed bring about substantial commitment to global health issues, it is important to note that the way HIV/AIDS was framed had and continues to have (often negative) implications for the sustainability and success of the program. It is important to note that HIV/AIDS was and continues to be a critical global health issue, but framing it as a security issue was limiting.^d^ Feldbaum notes that framing HIV/AIDS as a security threat was challenged over time where the questioned credibility of the security threat of the disease created barriers to the actual program that was developed to address the disease [21]. What needs to be asked is whether security is the most appropriate frame to improve global health and perhaps more importantly, is national security ethically justified when working towards global health in an interconnected world?

Philosophically, the security perspective contends, whether implicitly or explicitly, even more than the charity frame, that the strongest identity is that of nation-state. Subsequently, this state-based identity is considered the locus of responsibility. In other words, the national polity is responsible to further the good of the demos or the nation in this case. The lines of responsibility are demarcated according to formal citizenship. This logic has provided fodder for a strongly asserted opposition to a cosmopolitan ethic of *global* justice. This opposition draws on the fact that there is currently a lack of a global demos or citizenship tied to the lack of a global (supranational) authority [22]. Some suggest that without a global polity or a global rule of law for which to bind citizens and the reciprocal responsibility of citizen-state relationships that they engender, there is no basis for a transnational global ethic [23]. As was mentioned at the outset of this paper, this argument represents what Beitz calls the “morality of states” [4]. This outlook sees states as the “principle bearers of rights and duties rather than persons” and that these states are then “obligated to follow a system of norms analogous to those that apply to individuals in the state of nature” [4]. Ruger notes that within the state-centric ethical frames the “global health inequalities have no moral standing: justice, an associative obligation, is owed only to a government’s own citizens” (p. 428) [24].

Although the security frame shares the charity frames’ emphasis on responsibility to a national citizenship, it is distinguished by the tendency to motivate long-term action. The security frame follows the logic that sustained support for resource-poor regions or for collaborative forums will ensure sustained national security through enhanced diplomatic relations, good will fostered among the citizens of those regions (e.g. strategic war on terror) and the establishment of dependence and control [9]. For example, Brennan and Waldman describe the response to the earthquake that struck northern Pakistan and India in 2005, and note that “when natural disasters occur in countries in which the United States believes it has a national-security interest, a strong case can be made for long-term involvement” [25]. This statement is meant to demonstrate that security interests can actually provide long-term support for health issues in “foreign” countries, making it a good thing when compared to the reliance on short-term relief provided through “altruism” (characteristic of the charity frame) [25]. However, despite the good it produces, one must again ask, who is being left out because they do not engender security-based concerns? For example, the Global Health Security Initiative that was initiated by the United States provides a forum for countries to communicate and work to protect themselves from “key risks to global health security” including “chemical, biological, radiological and nuclear threats and the spread of pandemic influenza” [26]. Although this initiative is an important forum to address the key identified risks, the structure represents a common tendency towards strategic rather than global partnerships. The initiative, although linked to the World Health Organization, includes only the European Commission and eight other countries (only Mexico is represented from the Global South).

Despite the tendency to engender long-term health measures, the security frame is not consistent in this regard. Rubenstein provides the most nuanced critique of the security frame to date by articulating its underlying assumptions and analyzing whether these assumptions are in fact correct [27]. Rubenstein provides examples of how the following three desired assumptions, “health interventions contribute substantially to achieving objectives like (1) increasing security, (2) securing the allegiance of the population, or (3) stabilizing a region”, often do not result from “instrumental” uses of health interventions [27]. He refers to two studies that demonstrate that health interventions carried out by the United States military in the destabilized countries of Kenya and Afghanistan were seen as counterterrorism efforts rather than acts of concern for the local populations and thus did not have the desired effect of garnering allegiance or even achieving health benefits. Rubenstein discusses a recent case in which the CIA conducted a vaccination program in Pakistan in order to acquire blood samples and confirm the location of Osama Bin Laden, probably the most dramatic example of a health “ruse” to achieve national security objectives. This example represents the extreme end of “health instrumentalism” by highlighting that health was not even a desired outcome, demonstrated by the fact that follow-up vaccinations were not provided once the raid on Bin Laden’s compound was complete.

Frist affirms that the charity discourse has been replaced by that of “self-protection” despite the faith he places in health as a tool for international peace [14]. He notes that “health is a source of the most potent of forces in each human: the fear of death and the desire to preserve our own lives and the lives of those we love. Because health is so fundamental to all humans – of all nations, religions, races and situations – healthcare communicates a remarkable message of understanding and human connection across all boundaries and thus provides a unique, heretofore under-applied, tool of diplomacy” (p. 219) [14]. The aspirations that Frist puts forward are indeed noble and his thesis provocative, however, the examples provided above provide reason for concern. The limitation of the security frame, as demonstrated by the previous examples, is that trust, solidarity, and diplomacy are built on a foundation of altruism and mutual caring. With respect to the security frame, governments may not build trust but rather engage in superficial cooperation for their own self-interest [19]. The security frame may produce temporary and periodic global health benefits, but the examples provided in this section demonstrate that the ceiling of cooperation, global solidarity and system development is low.

### Cosmopolitanism

The final perspective is cosmopolitanism. Cosmopolitanism is founded on the following core principle.

“Cosmopolitanism takes the individual to be the ultimate unit of moral worth and to be entitled to equal consideration regardless of her culture, nationality or citizenship, besides other morally arbitrary facts about her (p. 431) [28].”

The cosmopolitan perspective contrasts the three perspectives described above, and particularly the security frame, by treating the individual as the ultimate unit of concern, a concern that takes primacy ahead of other units of identity such as nation and community. Thomas Pogge argues that “rich nations” have a negative duty to global citizens, those situated outside of one’s own borders, because of their role in creating and perpetuating a global order that often creates the conditions that are detrimental to the needs of those citizens [29]. This negative duty requires these nations not to cause harm to global persons through global economic and political practices. Pogge explicitly situates his argument for harm avoidance against beneficence or charity which do not acknowledge the systemic contributions to injustice and inequality. Gilabert critiques Pogge’s cosmopolitan project by articulating a positive duty to provide “reasonable assistance securing the conditions of autonomous agency” [30]. Gilabert argues that this positive duty promotes a solidarity that actually engages with a global citizenship to help create the conditions for human flourishing. In sum, the cosmopolitan account is supported by principles of harm avoidance and active assistance (positive solidarity) toward individuals at the global level over and above national allegiances characteristic of the above three forms of engagement.

The principles of individual moral worth and the duty not to harm and contribute to the betterment of the lives of individuals is then applied to the political sphere to ensure that justice “ought to apply among individuals *across* national boundaries, and not be limited within or constrained by these boundaries” (p. 431) [28]. The cosmopolitan ethical frame is inherently transnational in scope. The following section will review arguments that suggest that this ethical frame is the right one for a health-based foreign policy, that it engenders various goods that are not possible within a charity or security frame, and that a cosmopolitan frame is indeed possible.

### Why cosmopolitanism?

#### It is right

In the following section, I will present the reasons why the cosmopolitan ethical frame is morally right (versus the security and charity frames). To better understand the strengths of the cosmopolitan ethical frame it is beneficial to briefly discuss the purported weaknesses. David Miller provides the most comprehensive and nuanced argument against cosmopolitan justice and is specifically directed against a ‘global equality of opportunity principle’ [31] that asserts that individuals of equal capabilities should be able to achieve the same goals whether they are born in Canada or Afghanistan. Global justice in this sense strives to provide equal access to resources, thus affording equal opportunity to flourish or reach highest degree of individual potential in ones environment [32]. Miller presents a nuanced argument against the *global* equality of opportunity principle on numerous grounds including inherent differences between nations, that cosmopolitanism does not advocate for a particular political structure, yet the equality principle seems to advocate for a specific form of resource distribution and subsequently a specific form of governance, and that whether or not we live in a world of interconnectedness we still maintain a ‘special relationship’ (e.g. we owe more to our family relations than we do to our community) to our fellow citizen [31]. Despite Miller’s criticism of the ‘equality principle’, he remains committed to advocating for global justice, but in terms of power not equality. He maintains that the nation-state is best arbitrator of justice and that “democratically governed nations … are likely to make policy decisions that affect the resources and opportunities available to future generations of their own members, so that even if we were to imagine starting out from a baseline of equality, that equality will immediately be broken political and cultural differences between nations find expression in the policies that they pursue” (p. 71) [31]. Freeman also opposes the cosmopolitan emphasis on *global* justice, asserting that the state is the only sovereign system of politics in our contemporary political environment and thus the site of distributive and other forms of justice [33].

To demonstrate that it is indeed more “right” than the other perspectives I will review two core arguments in favor of cosmopolitanism. The first argument responds to the argument that special responsibilities to those “near and dear”, such as fellow citizens, are not contradictory to the cosmopolitan ideal, but rather a tension housed within the ideal itself [34]. This response is directed at a common criticism of cosmopolitanism that questions whether one can have a special responsibility to a particular relationship without de-valuing another relationship – directed at the cosmopolitan principle of general duty to all. To clarify, this particular opposition views national identity and the special responsibilities and relationships that this entails in direct contradiction of an ethic that extends responsibility to a “world citizenship” [35]. Abizadeh and Gilabert argue that although special relationships (non-instrumentally valuable) do indeed exist, that these relationships are only one “good” among many. They put forward a “conditionality thesis” that asserts that although certain relationships are non-instrumentally valuable, *all* relationships are conditionally valuable. They note that a relationship is dependent on human well-being (one good) and one may have special relationships (non-instrumentally value relationships – another good) that one’s special relationships may or may not give rise to special responsibilities depending on whether this relationship is conducive to ones well-being. For example, if a relationship harms one’s well being then they owe less to this relationship than one whereby their well-being is fostered. Abizadeh and Gilabert illustrate that one may have special relationships but remain committed to the anchoring principle of cosmopolitanism – that *every* human being has equal moral worth and subsequently that *every* individual (or state) must ensure this moral worth is protected and promoted irrespective of lesser allegiances such as nationality [34]. In other words, ones special responsibilities to those “near and dear” must be weighed against broader duties, both negative and positive, to uphold and ensure the basic rights of others. This logic suggests that the general duty to all persons within the cosmopolitan frame can foster the betterment of *global* health even if lesser allegiances, to say the health of persons within ones nation, are to be maintained. This logic is foundational to the human rights movement that establishes broader commitment to persons whether they are within politically bounded states or transnational.

The second argument is directed at the assertion that the “principles of justice should be applied within states” and that “the state … has normative significance as a context of justice” (p. 487) [36]. Caney provides three reasons ^e^ why this “statist scope thesis”, the thesis that justice is the business of states and their citizens, is unfounded but goes on to provide four ways in which the state indeed has normative significance not contrary to cosmopolitanism but *within* the cosmopolitan perspective itself (See Table 2). For example, he argues that states have instrumental importance (i.e. they are important as a *means* to particular ends) (importance applied to political institutions in general) in the pursuit of cosmopolitan policies, which is applicable to the foreign policy of countries and in the development of cosmopolitan institutions such as the World Health Organization that has embedded in its constitution the commitment to “health for all”. Caney returns to topic taken up by Abizadeh and Gilabert above by suggesting that the state has the responsibility and the capacity to serve the special duties to citizens as well as the general duty to global persons. The idea that the state itself can further cosmopolitan objectives aligns with contemporary understanding of foreign policy for health that recognizes that, far from being obsolete, states have an important role in fostering global health through foreign policy [8,37,38].

**Table 2:** Caney’s four points about the importance of the state within a cosmopolitan ethical frame

**Importance of the state**	**Role**
**Instrumental Contributions**	• They can pursue cosmopolitan policies (e.g. debt relief)
	• They can construct cosmopolitan institutions (e.g. World Health Organization)
**Upholding Rights**	• Institutional arrangements to uphold persons rights within and outside of state borders
**Engendering Justice**	• A national identity can rally support for state policies that uphold cosmopolitan ethics
**Locus of Compensation**	• Provide compensation for global injustice (e.g. compensation for state enacted injustices such as colonization – Britain may provide compensation to Indian citizens for injustices enacted during colonial rule)

The above arguments in favor of a cosmopolitan ethic underscore the perspective that 1) the state (even if instrumentally valuable for the organization of health services) is a morally arbitrary unit of relationships and responsibility (when compared to the less arbitrary unit of moral value, the human being) and 2) that the state and cosmopolitan global justice are not mutually exclusive.

Why is it then that the cosmopolitan ethical frame is *more* right than the security or charity frames? The first reason is that, in an interconnected world order, the morality of states as the *primary* unit where justice is concerned is insufficient to address transnational health issues. The reality of our interconnected world is that the choices and actions of those in one country influence the conditions in another. The very existence of the field of global health indicates that the determinants of health and the capacity to address the health of persons are transnational. Scholars of global health recognize this [12,39]. However, one could argue that global health goals can be achieved through security and charity perspectives. Drawing on the above arguments, cosmopolitanism can be thought of as a higher order ethical framework than these two frames. The emphasis on *persons* rather than nations extends the ethical scope of foreign policy to include *all* individuals. In this sense, the motives of security are addressed, the responses resulting from the charity frame are maintained, but the scope of moral concern is expanded to include 1) persons and 2) issues such as non-communicable diseases, which may not have as strong a security component as the transmission of infectious diseases. For example, transnational cooperation to address Cancer in Bolivia does not have direct security implications for a country like Canada as much as a SARS outbreak in Bolivia would. The cosmopolitan frame provides an ethical argument for Canada to care about and distribute resources if necessary to address this issue.

The second argument for the *rightness* of a cosmopolitan frame is rooted in the nature of the state. Even the opponents of a cosmopolitan ethic of global justice recognize the arbitrary nature of the state when compared to the identity of being human [31]. The UN Declaration of Human Rights reflects the arbitrariness of the state by enshrining the principle that the individual is the ultimate unit of moral worth and not the state. For example, numerous humanitarian interventions have taken place to protect the basic rights of individuals living within tyrannical states. In such cases, the rights of states (e.g. sovereignty and autonomy) may be transgressed by an international community initiative to protect the rights of its citizens. This phenomenon is not new. Philosophically, the human identity precedes the identity of state citizenship. Another way to present this argument is through the following rationale. If I am born in Canada, I automatically adopt all of the political, social and economic benefits of citizenship. If I choose to move to Afghanistan I am free to do so. I may then wish to apply for citizenship in Afghanistan. If this citizenship is granted, I may choose to release myself from my association to Canada and my formal citizenship in this country. In legal terms, I am now Afghani but remain a human being. My humanness is proven less arbitrary than my citizenship. In fact I am the same human being that I was when I was a Canadian citizen. It is exactly this logic that a cosmopolitan frame is founded on and subsequently the same logic that the human rights discourse has used to protect and promote the rights of the “other”.

To summarize, I have presented common criticisms of cosmopolitanism and their counter-arguments. I have then brought this discussion back to the purpose of this paper, which is to argue for a cosmopolitan ethical frame to undergird foreign policy for health. I argue that there are two reasons why this cosmopolitan frame is more right than a security or charity frame, namely that the emphasis on *persons* engendered by cosmopolitanism is more fundamental than a morality of states. The following section argues that the cosmopolitan ethical frame is *good* for a states foreign policy.

#### It is good

The sovereignty of states is one commonly unquestioned good that must be weighed against the good of human flourishing [40]. The good of state sovereignty is important when various transnational interests seek to undermine health-protective state policy [41]. However, the other side of sovereignty is a preoccupation with independence amidst interdependence. This preoccupation can border on isolationism or inter-state antagonism that is so detrimental to stability in global politics. This type of sovereignty underlies countries’ resistance to deep levels of international integration in order preserve perceived national benefits. In an article about access to vaccines for H5N1 and H1N1 viruses and the difficulty of the diplomatic process that surrounded this case, Fidler concludes that “states have not agreed to binding agreements on more equitable access but, rather, attempt to increase such access through ad hoc, reactive, and nonbinding activities that preserve national freedom of action while demonstrating some humanitarian concern” [42]. The anarchic nature (i.e. lack of supranational/global authority) of the global political order requires countries to engage or not engage with other countries on a voluntary basis to achieve goals of global scope [43]. Labonte reminds us that “states, the people who govern them and the institutions they create are moral actors not exempt from a capacity for, and necessity of ethical justification for their actions” [9]. The ethical justification of security is limited in achieving global public goods such as the Framework Convention for Tobacco Control (FCTC). It should be noted that vision or intention (i.e. security vs. cosmopolitan justice) is necessary but not sufficient for action on global health issues. Ethical perspectives can orient political action to particular ends and to the development of particular means to achieve those ends, but it would be naïve to assert that such perspectives eliminate “real world” barriers to realization. Abizadeh eloquently states this point when he asserts that “a shared global identity may face many obstacles, but metaphysical impossibility and conceptual confusion are not among them”. The FCTC is one example where concern for global health resulted in a binding legal tool to guide the scaling up of national legislation for health protection and disease prevention [44]. Unfortunately, the low-politics of health continues to confront the high-politics of economics and security [15,38]. The relationship between trade and health with respect to tobacco has created a vigorous debate and an active legal environment where countries continue to be challenged within trade and investment treaties as they attempt to implement provisions of the FCTC to improve health [45,46]. This is not to say that trade and health cannot be mutually beneficial [47]. The possibility of mutual benefit is precisely one of the projects for those working to make foreign policy better for global health. The project has many core objectives but coherence remains at the center. One of the goods that a cosmopolitan ethical frame can contribute is a lens to view intersectoral (e.g. health, trade, investment) coherence in foreign policy. For example, the cosmopolitan frame may prompt one to ask, “Does Canada’s engagement within the contemporary trade and investment regulatory environment contribute to poorer health in a country like Bolivia? In the long-term, does this trade contribute to non-communicable disease in Bolivia? If so, are their ways that the two countries can negotiate a better arrangement for the health of Bolivia’s population beyond the simple economic interests of Canada?” The central point is that the cosmopolitan frame allows for the consideration of health beyond simply short-term health threats and national security.

The cosmopolitan frame also engenders the good of long-term systemic commitment towards global health goals. Some have criticized cosmopolitanism for requiring institutional change while claiming to be agnostic to institutional design [31]. However, many scholars of cosmopolitanism are not agnostic to an institutional development project. For example, David Held has written extensively on the possibility of supranational institutions to facilitate a global democratic process [48,49]. Caney has also developed core principles for supranational institutions for the facilitation of global justice [50]. I suggest that what a cosmopolitan ethical frame engenders, more than the charity and security frames, is a vision for transnational cooperation and institution *building*. This institutionalization may take the form of a global taxation system to redistribute funds to less-well-off countries [29,51] or a global fund to scale-up human resources for health. Whatever form this transnational governance takes, the adoption of a cosmopolitan frame represents the first step. The point being asserted here is that national self-interest is more likely to withhold full participation in transnational endeavors because the state-based identity does not lend itself to a transnational identity or self-sacrificial cooperation.

Countries are familiar with treaty and regulation development at the global level. However, even when such tools are created, the implementation phase is plagued by a lack of institutional or financial support [52]. This plague is directly tied to a model of national self-interest. It is harder to release domestic resources to global ends when doing so does not directly benefit the contributing country. For example, it was mentioned previously that the NCDs caused by tobacco are not a direct security threat to Canada, so Canada would need to rely on a charity frame to contribute to FCTC implementation in another country. Gostin notes that “if assistance is always tied to self-interest, then funding will always be skewed toward what the rich want to deliver, rather than toward the larger, systemic problems of the poor” (p. 10) [53]. Continuing with the above example, from the perspective of the charity ethical frame Canada may be less likely to channel resources into sustainable system building over programs that produce immediate and tangible results that can be demonstrated to their stakeholders. The Global Fund to Fight AIDS, Malaria and TB is one example of how a foreign policy of collaboration can result in a transnational system of governance. It is noteworthy that the apparatus of the Global Fund appears to reflect the cosmopolitan ideal of transnational justice, but the Fund itself has recently struggled to replenish its funds. This struggle does not only stem from a lack of a cosmopolitan ethical perspective by donors but also engendered mistrust based on mismanagement and abuse of funds by recipient countries.

Despite the “real world” challenges of global funding streams for transnational health initiatives, it is still fair to assert that if states and those working in the health field frame their work in cosmopolitan terms it is more likely that global health goals are to be sustainably financed (whether in the form of global funds, or other governance mechanisms) and will more likely provide sustain benefits to those in need. To achieve sustainability Brock argues for a “needs-based minimum floor principle” [51]. This principle supports the provision of minimum standard of sustenance for all, rather than channeling resources to the least well off. This standard would require international consensus, similar to the consensus required by international treaties, and be adapted to the different national contexts, but the ideal behind this principle is that persons would have access to a basic standard of living no matter what country they live in, while affording individual discretion to determine the ceiling of wealth [51]. Many countries incorporate this principle when instating social safety nets and economic opportunities; however this has not yet been undertaken as a global project. This principle is important for a healthy foreign policy in that it is widely recognized that many of the determinants of health fall outside of the formal health systems of countries (e.g. income). Horton suggests that, “health moves foreign policy away from a debate about national interests to one about global altruism” [54]. Although altruism is an important component of foreign policy for health, the good that a cosmopolitan frame engenders is a *duty* and *responsibility* to all persons and the creation of systems to facilitate this.

#### It is possible

To what extent is the cosmopolitan ideal possible in this contemporary economic and political environment? Would countries be able to contribute *scarce* resources to cosmopolitan initiatives to improve global health? This section will argue that the current economic and political orders are not *natural* barriers to such endeavors. This argument will confront the possible assertion that the cosmopolitan project is utopian and impractical amidst an environment of resource limitations and state-based distribution practices. Schrecker makes a persuasive argument against the *natural* occurrence of resource scarcity [55]. One theme of his argument is that particular (perceived) scarcities are in fact a result of political decisions. For example he refers to the findings of the Bellagio Study Group that estimate that “a package of interventions costing US$ 5.1 billion per year would save the lives of 6 million children per year in 42 countries that account for 90% of the global toll of under-5 child mortality” (p. 601) [55]. To put this number in perspective, in 2007 the Canadian GDP was US$ 1.3 trillion dollars [55]. The resources are in fact available to improve global health and it is only when the scarcity of these resources is “de-naturalized” that governments can begin to reorient their distribution practices. This argument is supported by Pogge’s argument that “rich” governments are actually complicit in the state of global injustices, by developing intergovernmental initiatives that favor their own self-interest, supporting the transfer of funds to corrupt governments, among other things [29].

A subtle yet important dimension of the scarcity argument is that much of the “scarcity” is a result of the self-interested frames adopted by states. This self-interest is a significant motivation for holding back resources for fear that they will not be invested in ways that will directly benefit the paying country. The shortfalls among UN agencies is one example of this phenomenon where there is reluctance to fund organizational work and to provide funds for specific, tangible, projects. Again this ties back to our previous argument that a cosmopolitan ethical frame can provide the impetus for system building and long-term planning. It is important to note that Schrecker observes that the many that call for the global redistribution of resources view it as untenable within the current state-orient framework [55]. He counters this view unambiguously by stating that “indeed, it is perverse in the extreme to reject the existence of health-related ethical obligations that cross national borders simply *because* no mechanisms exist to hold powerful social institutions, and the key actors within them, accountable for scarcities they cause or perpetuate, perhaps half a world away” (p. 603) [55].

## Summary

Foreign policy has great potential to improve the state of global health. This field of research and practice recognizes the importance of states in fostering global health goals. However, it is hoped that this paper will facilitate a reflection and dialogue on the implications of current charity and security discourses are morally grounded and whether they achieve their desired effect. I recognize that states are bound to protect and promote the health of their citizens and this impels them to play a “two-level” game in international forums [56] a game that at once negotiates the tension between domestic politics and global objectives. Schieber and colleagues conclude their analysis and critique of the current systems of financing global health by stating that: “Donor countries will need to better balance national political considerations with global health needs and work together to achieve results” [3]. I would add that the donor-recipient structure has its own inherent problems (including the responsibility of countries to use funds appropriately for the benefit of persons), but this statement lies at the crux of this paper. The question is whether independent states can move towards global health goals while operating within an ethical framework that prefers “national political considerations”, such as security. This paper has argued that an ethical frame must inspire and motivate countries to move beyond state interests to consider the needs of a global citizenship. This argument is far from utopian and can find its roots, if not its practical considerations, in the Universal Declaration on Human Rights. This international norm is one that recognizes the rights of persons over a “morality of states”. This paper suggests that global health goals must be motivated by a *global* ethic of justice and that the ethical frames of charity and security in fact dissuade countries, whether intentionally or unintentionally, from creating the “goods” of transnational system-building, long-term planning and implementation, and systematic cooperation and coordination.

Examining the ethical perspectives motivating foreign policy for health is an important development in both the scholarly work it produces and the practice that it engenders. I have described the ethical state of affairs in this field and note that the charity and particularly the security ethical perspectives remain dominant. I further argue that these ethical perspectives are morally and practically weak when held up against a cosmopolitan ethical frame. The cosmopolitan ethical frame can provide a heuristic for the analysis and implementation of foreign policy for health. This frame is not only morally right, but can engender specific goods that would not be as likely in its absence, and is possible from a resource perspective. Indeed foreign policy has great potential to improve the health of global persons. The cosmopolitan ethical frame can serve to realize this potential.

### Endnotes

^a^In this case I am referring to state interests such as the prevention of war, which is a collective threat based on ideological differences, boundary disputes or other *political* rationale. I recognize that issues such as war have a direct impact on human health (in fact this is one of the most dramatic assaults to health) but the experience of health (an individual phenomenon) is distinguishable from the conditions that influence health (social, economic and political phenomenon).

^b^I recognize that this perspective is different than a population health perspective that views health in the aggregate.

^c^It is true that the treatment of chronic disease has implications for state-run/funded public health care systems, however these implications are only indirect security threats.

^d^For example, approximately 2.4 million people are being supported by the United States PEPFAR program (notably, a small portion of those with the disease).

^e^Caney argues that this thesis is 1) morally arbitrary, 2) incomplete and 3) theoretically inadequate to deal with *transnational* issues.

## Competing interests

The author declared that he has no competing interests.

## Pre-publication history

The pre-publication history for this paper can be accessed here:

http://www.biomedcentral.com/1472-698X/13/29/prepub

## References

[B1] WHO World Health Statistics 2011 2011 Geneva

[B2] Waage J Banerji R Campbell O Chirwa E Collender G Dieltiens V Dorward A Godfrey-Faussett P Hanvoravongchai P Kingdon G The millennium development goals: a cross-sectoral analysis and principles for goal setting after 2015: lancet and london international development centre commission Lancet 2015 2010 376 991 1023 10.1016/S0140-6736(10)61196-8 PMC7159303 20833426

[B3] Schieber GJ Gottret P Fleisher LK Leive AA Financing global health: mission unaccomplished Health Aff 2007 26 921 934 10.1377/hlthaff.26.4.921 17630434

[B4] Beitz C Brock G, Moellendorf D Cosmopolitanism and global justice Current Debates in Global Justice. Volume 2 2005 Netherlands: Springer 11 27 Studies in Global Justice

[B5] Kickbusch I Silberschmidt G Buss P Global health diplomacy: the need for new perspectives, strategic approaches and skills in global health Bull World Health Org 2007 85 230 232 10.2471/BLT.06.039222 17486216 PMC2636243

[B6] Drager N Fidler DP Foreign policy, trade and health: at the cutting edge of global health diplomacy Bull World Health Org 2007 85 162 162 10.2471/BLT.07.041079 17486200 PMC2636230

[B7] Gostin LO Archer R The duty of states to assist other states in need: Ethics, human rights, and international law J Law Med Ethics 2008 35 526 533 18076505 10.1111/j.1748-720X.2007.00177.x

[B8] Labonte R Schrecker T Foreign policy matters: a normative view of the G8 and population health Bull World Health Org 2007 85 185 191 10.2471/BLT.06.037242 17486209 PMC2636225

[B9] Labonte R Gagnon ML Framing health and foreign policy: lessons for global health diplomacy Globalization Health 2010 10.1186/1744-8603-1186-1114 PMC2936293 20727211

[B10] Merchant RM Leigh JE Lurie N Health care volunteers and disaster response — first, Be prepared N Engl J Med 2010 362 872 873 10.1056/NEJMp1001737 20181966

[B11] Schwartz D Michaëlle Jean: 'You cannot build a sustainable economy on charity' 2012 Ottawa: CBC News

[B12] Labonte R Mohindra K Lencucha R Framing international trade and chronic disease Globalization Health 2011 7 21 10.1186/1744-8603-7-21 21726434 PMC3158109

[B13] Dowell SF Tappero JW Frieden TR Public health in Haiti — challenges and progress N Engl J Med 2011 364 300 301 10.1056/NEJMp1100118 21219131

[B14] Frist WH Medicine as a currency for peace through global health diplomacy Yale Law Policy Rev 2007 26 209 229

[B15] Feldbaum H Michaud J Health diplomacy and the enduring relevance of foreign policy interests PLoS Med 2010 7 e1000226 10.1371/journal.pmed.1000226 20422036 PMC2857879

[B16] Clinton HR President’s Global Health Initiative 2009 Washington: Department of State

[B17] Kellerhals MD Obama proposes massive global health initiative 2009 Washington: US Government

[B18] US Department of Health and Human Services The global health strategy 2011 Washington

[B19] Aldis W Health security as a public health concept: a critical analysis Health Policy Plann 2008 23 369 375 10.1093/heapol/czn030 18689437

[B20] Katz R Kornblet S Arnold G Lief E Fischer JE Defining health diplomacy: changing demands in the Era of globalization Milbank Q 2011 89 503 523 10.1111/j.1468-0009.2011.00637.x 21933277 PMC3214719

[B21] Feldbaum H U.S. Global Health and National Security Policy: A report for the CSIS Global Health Policy Center 2009 Washington: Center for Strategic and International Studies

[B22] Archibugi D Cosmopolitan democracy and its critics: a review Eur J Int Relations 2004 10 437 473 10.1177/1354066104045543

[B23] Baker G Problems in the theorisation of global civil society Political Studies 2002 50 928 943 10.1111/1467-9248.00401

[B24] Ruger JP Health, capability, and justice: Toward a new paradigm of health ethics, policy and law Cornell J Law Pub Policy 2005 15 403 482 17136814

[B25] Brennan RJ Waldman RJ The south Asian earthquake Six months later — an ongoing crisis N Engl J Med 2006 354 1769 1771 10.1056/NEJMp068017 16641392

[B26] Global Health Security Initiative: 10 years of collaborative action 2011 Brussels: European Commission

[B27] Rubenstein LS Instruments of peace: The use of health for national security Harvard Int Rev 2011 33 46 50

[B28] Tan K-C Liberal Nationalism and Cosmopolitan Justice Ethical Theory Moral Prac 2002 5 431 461 10.1023/A:1021339410934

[B29] Pogge T World poverty and human rights Ethics Int Affairs 2005 19 1 7

[B30] Gilabert P The duty to eradicate global poverty: positive or negative? Ethical Theory Moral Prac 2005 7 537 550 10.1007/s10677-005-6489-9

[B31] Miller D Brock G, Moellendorf D Against global egalitarianism Current Debates in Global Justice. Volume 2 2005 Netherlands: Springer 55 79 Studies in Global Justice

[B32] Ruger JP Global health justice Pub Health Ethics 2009 2 261 275 10.1093/phe/php019

[B33] Freeman S Justice and the social contract 2007 New York: Oxford University Press

[B34] Abizadeh A Gilabert P Is there a genuine tension between cosmopolitan egalitarianism and special responsibilities? Philosophical Studies 2008 138 349 365 10.1007/s11098-006-9046-z

[B35] Nussbaum MC Kant and Stoic Cosmopolitanism J Politic Philosophy 1997 5 1 25 10.1111/1467-9760.00021

[B36] Caney S Global distributive justice and the state Political Studies 2008 56 487 518 10.1111/j.1467-9248.2008.00748.x

[B37] Bettcher DW Yach D Guindon GE Global trade and health: key linkages and future challenges Bull World Health Org 2000 78 521 534 10885181 PMC2560735

[B38] Fidler DP The globalization of public health: the first 100 years of international health diplomacy Bull World Health Org 2001 79 842 849 11584732 PMC2566654

[B39] Fidler DP Health as foreign policy: harnessing globalization for health Health Promotion Int 2006 21 51 58 10.1093/heapro/dal051 17307957

[B40] Ruger JP Health capability: conceptualization and operationalization Am J Public Health 2009 100 41 49 19965570 10.2105/AJPH.2008.143651 PMC2791246

[B41] Lencucha R Philip Morris versus Uruguay: health governance challenged Lancet 2010 376 852 853 10.1016/S0140-6736(10)61256-1 20833287

[B42] Fidler DP Negotiating equitable access to influenza vaccines: global health diplomacy and the controversies surrounding avian influenza H5N1 and pandemic influenza H1N1 PLoS Med 2010 7 e1000247 10.1371/journal.pmed.1000247 20454566 PMC2864298

[B43] Wendt A Anarchy is what states make of it: the social construction of power politics Int Org 1992 46 391 425 10.1017/S0020818300027764

[B44] Roemer R Taylor A Lariviere J Origins of the WHO framework convention on tobacco control Am J Public Health 2005 95 936 938 10.2105/AJPH.2003.025908 15914812 PMC1449287

[B45] Mitchell A Voon T Implications of the World Trade Organization in combating non-communicable diseases Public Health 2011 125 832 839 10.1016/j.puhe.2011.09.003 22030320

[B46] Taylor A Chaloupka FJ Guindon E Corbett M Jha P, Chaloupka FJ The impact of trade liberalization on tobacco consumption Tobacco control in developing countries 2000 New York: Oxford University Press

[B47] McGrady B Trade and tobacco control: Resolving policy conflicts through impact assessment and administrative type international laws Asian J World Trade Org Int Health Law Policy 2008 3 341 378

[B48] Held D Democratic accountability and political effectiveness from a cosmopolitan perspective Gov Opposition 2004 39 364 391 10.1111/j.1477-7053.2004.00127.x

[B49] Held D Law of states, law of peoples Legal Theory 2002 8 1 44 10.1017/S1352325202081016

[B50] Caney S Cosmopolitan justice and institutional design: An egalitarian liberal conception of global governance Soc Theory Prac 2006 32 725 756

[B51] Brock G The difference principle, equality of opportunity, and cosmopolitan justice J Moral Philosophy 2005 2 333 351 10.1177/1740468105058158

[B52] Magnusson RS Global health governance and the challenge of chronic, Non-communicable disease J Law Med Ethics 2010 38 490 507 10.1111/j.1748-720X.2010.00508.x 20880237

[B53] Gostin LO International development assistance for health: Ten priorities for the next president Hastings Cent Rep 2008 38 10 11 18947130 10.1353/hcr.0.0061

[B54] Horton R Health as an instrument of foreign policy Lancet 2007 369 806 807 10.1016/S0140-6736(07)60378-X 17350433

[B55] Schrecker T Denaturalizing scarcity: a strategy of enquiry for public- health ethics Bull World Health Org 2008 86 600 605 10.2471/BLT.08.050880 18797617 PMC2649456

[B56] Putnam RD Diplomacy and domestic politics: the logic of two-level games Int Org 1988 42 427 460 10.1017/S0020818300027697

